# Langya virus outbreak in China, 2022: Are we on the verge of a new pandemic?

**DOI:** 10.1016/j.jve.2022.100087

**Published:** 2022-09-17

**Authors:** Shehroze Tabassum, Aroma Naeem, Syeda Tayyaba Rehan, Abdulqadir J. Nashwan

**Affiliations:** King Edward Medical University, Lahore, Pakistan; Dow University of Health Sciences, 74200, Karachi, Pakistan; Hamad Medical Corporation, Doha, Qatar

**Keywords:** Langya, Outbreak, Infectious disease, LayV, Langya henipavirus, HeV, Hendra virus, NiV, Nipah virus

Dear Editor

Langya henipavirus (LayV) is an emerging pathogen that belongs to the family Paramyxoviridae. It has been recently widelys related to the Hendra (HeV) and Nipah (NiV) species, the latter having a high fatally rate^1^ and are known to be able to infect both humans and animals. LayV belongs to *the Paramyxovridae* family.^2^ Zoonotic HeV has its origin in Australia and infects horses, while NiV has an animal reservoir in fruit bats and has caused multiple outbreaks in South East Asia.^3^ LayV genome comprises 18,402 nucleotides.^4^ It is a zoonotic pathogen; in the vicinity of human cases, the rate of LayV antibodies was 5% in dogs and 2% in goats.^3^ LayV RNA was isolated from 27% of 267 shrews (small mole-like animals),^2^ its main animal reservoir, suggesting that shrews are a possible reservoir for human transmission. However, the exact mechanism of its spread to humans is still unknown. The range of symptoms includes fever, fatigue, and cough^2^ to fatal encephalitis.

The first ever hint of LayV human transmission emerged in China when a 53-year-old farmer visited a hospital in Shandong province in December 2018 who presented with fever and a history of contact with animals within a month of symptom onset.^2^ The LayV genome, was detected in throat swabs of the aforementioned farmer upon enrollment into a screening study for zoonotic diseases. This study included 35 patients from 3 hospitals of Shandong and Henan provinces who were suspected to be infected by LayV.^5^ Results from this study show the presence of the LayV genome in 26 out of 35 of the study participants, suggesting that LayV was the sole cause of the illness.^6^ Out of these 26 cases, all had a fever, 50% a cough, 46% myalgia, 35% a headache, and vomiting was present in 35% of them. Fifity-four percent of them had leukopenia, 35% thrombocytopenia, with some showing abnormal liver function.^7^ Most of them were farmers and some factory employees,^3^ which suggests that LayV human infection bears a relationship with farmers' environmental exposure. When 15 close family contacts of 9 patients, were investigated there was not signs of infection,^3^ which suggests sporadic transmission in humans, however, the sample size was small to darw firm conclusions. No clusters of cases were found within the same family or geographical proximity.^2^ None of all patients infected with LayV in China have been reported to have died , so the fatality rate is still not totally clear.^7^ t.

The threat is that we are unaware of what these adaptations might be and what might these adaptations do and thus possess the potential to unlock several means of global spread. However, we can take a breath of relief as no human-to-human transmission has yet been reported.^8^ So, there are high chances that LayV cannot become a pandemic in contrast to COVID-19. Living in a world of pandemics like COVID-19 and monkeypox, we must keep close eyes on emerging infectious diseases like LayV. As little is known about the pathogenicity of LayV, further research should focus more on knowledge of viron to reach the root mechanism behind its infectivity and spread. Strategies for community-based surveillance should be developed to combat the LayV outbreak. Investigations should be carried out to know more about the exact mode of transmission; help can be sought from already discovered cousin viruses, HeV and NiV, to know more about LayV, its structure, and mode of spread to detect and promptly contain the disease timely. Planning of outbreak management strategies is the need of the hour as the emergence of LayV in eastern China suggests how effortlessly this virus can spread, unobserved, from animals to humans^5^ and thus possesses the potential to be a global threat. Out of 26 patients who tested positive for LayV, the intensity of disease was found to be severe in patients with compromised immunity.^9^ This emphasizes the need to focus on immunity boosters to prevent disease progression. Centers of disease control and prevention (CDC) suggest symptomatic management of complications with supportive care; however, trials in laboratory studies have shown beneficial results with antiviral ribavirin.^6^ We suggest that extensive research is necessary in the area of vaccines and antiviral treatments.

## Funding

No external funding has been received for this paper.

## Authors’ contributions

Conceptualization: STR, ST, Writing- Original draft: ST, AN, STR, Editing, Review- original draft: STR.

## Declaration of competing interest

The authors declare that they have no known competing financial interests or personal relationships that could have appeared to influence the work reported in this paper.

## References

[1] Marsh G.A., Wang L.F. (2012 Jun). Hendra and Nipah viruses: why are they so deadly?. Curr Opin Virol.

[2] Mallapaty S. (2022 Aug 11). New ‘Langya’ virus identified in China: what scientists know so far. Nature.

[3] Lu D. (2022 Aug 10). Newly identified Langya virus tracked after China reports dozens of cases. Guardian.

[4] Henipaviruses - aljofan - major reference works - wiley online library. https://onlinelibrary.wiley.com/doi/abs/10.1002/9780470015902.a0023275.

[5] CNN SM New virus found in China could be “tip of the iceberg”. https://www.cnn.com/2022/08/12/china/china-new-virus-disease-animal-spillover-intl-hnk/index.html.

[6] (2022). New concerning Langya virus identified in China—here’s what you need to know. Prevention.

[7] Five things you need to know about Langya virus. https://www.gavi.org/vaccineswork/five-things-you-need-know-about-langya-virus.

[8] News · LP C (2022). ANALYSIS | Why it's worth keeping “close eye” on new Langya virus that's infecting dozens in China | CBC News. CB (Curr Biol).

[9] US AC The conversation. What is the new Langya virus, and should we Be worried? [Internet], Sci Am. https://www.scientificamerican.com/article/what-is-the-new-langya-virus-and-should-we-be-worried/.

